# Exercise-Based Cardiac Rehabilitation for Peripheral Artery Disease

**DOI:** 10.3390/jcm15082826

**Published:** 2026-04-08

**Authors:** Francesco Giallauria, Mario Pacileo, Gianluigi Cuomo, Giuseppe Vallefuoco, Fabrizio Catalini, Crescenzo Testa, Cristina Savarese, Alfredo Mauriello, Carmine Izzo, Michele Ciccarelli, Vincenzo Russo, Antonello D’Andrea

**Affiliations:** 1 Department of Translational Medical Sciences, Federico II University of Naples, via S. Pansini 5, 80131 Naples, Italy; fabrizio.269@libero.it (F.C.); crescenzo.testa@unipr.it (C.T.); 2 Cardiology and Intensive Care Unit, Department of Cardiology, “Umberto I” Hospital, 84014 Nocera Inferiore, Italy; m.pacileo@aslsalerno.it (M.P.); c.savarese@aslsalerno.it (C.S.); antonellodandrea@libero.it (A.D.); 3 Department of General Medicine, “Ospedale del Mare”, ASL Napoli 1 Centro, 80147 Naples, Italy; gianluigi.cuomo@aslnapoli1centro.it; 4 Cardiology Unit, “V. Monaldi” Hospital, University of Campania “Luigi Vanvitelli”, 80131 Naples, Italy; giuseppevallefuoco46@gmail.com; 5 Cardiology Unit, Institute National Cancer, IRCCS, Foundation “G. Pascale”, 80131 Naples, Italy; alfredo.mauriello93@libero.it; 6 Department of Medicine Surgery and Dentistry “Scuola Medica Salernitana”, University of Salerno, 84084 Salerno, Italy; cizzo@unisa.it (C.I.); mciccarelli@unisa.it (M.C.); 7 Cardiology Unit, Department of Medical and Translational Sciences, University of Campania “Luigi Vanvitelli”, “V. Monaldi” Hospital, University of Campania “L. Vanvitelli”, 80131 Naples, Italy; vincenzo.russo@unicampania.it

**Keywords:** cardiac rehabilitation, exercise training, peripheral artery disease

## Abstract

Peripheral artery disease (PAD) is a pervasive atherosclerotic condition affecting well over 100 million adults worldwide and associated with major functional limitations, reduced quality of life, and elevated risks of myocardial infarction, stroke, limb events, and mortality. Exercise therapy—preferably supervised or delivered through structured, monitored home-based programs—is a first-line, guideline-endorsed therapy that improves walking performance and patient-reported outcomes and contributes to comprehensive secondary prevention. This review synthesizes mechanistic underpinnings (endothelial, angiogenic, metabolic, and autonomic) and appraises the comparative effectiveness, safety, and implementation models of supervised exercise therapy (SET), structured home-based and hybrid programs, and alternative modalities in PAD. Finally, we summarize policy aspects and persistent gaps to guide clinical practice and future research.

## 1. Introduction

Lower-extremity peripheral artery disease (PAD) is a chronic, occlusive manifestation of atherosclerosis, increasingly recognized as a heterogeneous condition encompassing multiple clinical and functional phenotypes, characterized clinically by intermittent claudication, rest pain, non-healing ulcers, or critical limb-threatening ischemia (CLTI) [1]. Diagnostic confirmation relies on a resting ankle–brachial index (ABI) ≤ 0.90, with toe pressures and transcutaneous oxygen measurements helpful in suspected CLTI [2].

However, beyond these classical manifestations, patients may present multiple phenotypes, with distinct clinical, functional, anatomical, biological and metabolic characteristics. Functional phenotypes are defined by reduced walking capacity and exercise tolerance, which can be present even in the absence of classical claudication, highlighting that limitations in mobility and quality of life may occur independently of symptomatic status [1]. Anatomical phenotypes, which are crucial for therapeutic decision-making, are often classified according to the TASC II (TransAtlantic Inter-Society Consensus) criteria, distinguishing lesions by location, length, and complexity [3]:

- Type A lesions: short, focal stenoses or occlusions, usually amenable to endovascular therapy;
- Type B lesions: slightly longer or multiple lesions, still generally treated percutaneously;
- Type C lesions: more extensive occlusions or complex patterns, where surgical bypass may be preferred;
- Type D lesions: diffuse, multisegmental disease, often requiring surgical intervention.

Recognition of these anatomical phenotypes helps predict functional limitation, exercise tolerance, and the likely response to rehabilitation interventions or revascularization strategies. Biological and genetic phenotypes reflect underlying molecular and inflammatory pathways, including variations in vascular remodeling, endothelial function, and susceptibility influenced by specific genetic polymorphisms, while metabolic phenotypes, such as metabolically healthy versus unhealthy obesity, contribute to differential risk and progression of PAD [4].

PAD confers high risks for major adverse cardiovascular events (MACEs) or major adverse limb events (MALEs) and impaired function even among patients without classic claudication. Contemporary multi-society guidelines (ACC/AHA 2024; ESC/ESVS 2024) re-affirm structured exercise as a core component of care, along with high-intensity statin therapy, smoking cessation, antithrombotic strategies, and individualized consideration of revascularization where indicated [2].

The global epidemiology of PAD points to an aging population, with gender, socioeconomic, and geographic disparities. The Lancet Global Health 2019 GBD assessment estimated ≈113 million people ≥40 years living with PAD in 2019. Newer analyses confirm high contributions from modifiable risk factors and rising total numbers despite heterogeneous age-standardized trends. These data underscore the need for scalable exercise solutions integrated with preventive therapies [5,6].

The heterogeneity of PAD phenotypes suggests that exercise-based rehabilitation should be considered within a patient-centered framework, taking into account differences in clinical presentation, functional capacity, and anatomical disease burden.

The goal of this review is to synthesize current evidence on exercise-based rehabilitation for PAD, integrating mechanistic insights, clinical effectiveness, safety, and implementation strategies, while highlighting gaps and future research priorities.

## 2. Methods

This narrative review was conducted using a structured literature search to identify relevant studies on exercise-based rehabilitation in peripheral artery disease (PAD).

**Figure 1:**
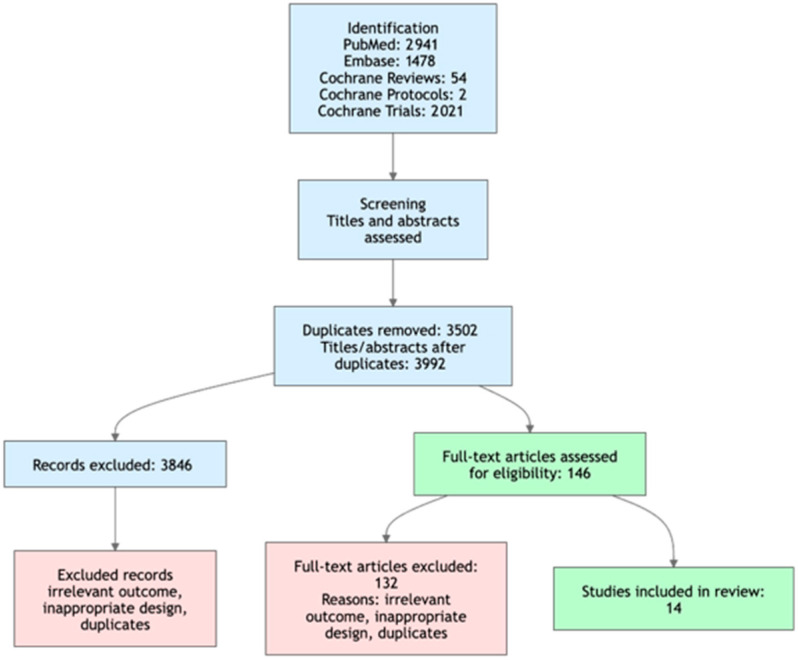
Search strategy.

A systematic search was performed in PubMed, Embase, and the Cochrane Library for articles published between 1 January 2010 and 31 January 2026. The search strategy included combinations of the following terms: (“peripheral artery disease” OR “PAD”) AND (“exercise therapy” OR “rehabilitation” OR “walking” OR “supervised exercise therapy” OR “home-based exercise”). The search strategy was adapted for each database using appropriate controlled vocabulary and keywords. In the Cochrane Library, broader terms such as “peripheral arterial disease” and “intermittent claudication” were used to account for differences in indexing and terminology (Figure 1).

Eligible studies included randomized controlled trials, meta-analyses, and major international guideline documents focusing on exercise interventions in patients with PAD. Observational studies were considered when providing complementary or real-world evidence. Case reports, non-clinical studies, and articles not published in English were excluded.

Study selection was based on relevance to the topic and methodological quality. Titles and abstracts were initially screened, followed by full-text assessment of selected articles. Emphasis was placed on recent and high-quality evidence, including contemporary randomized trials and meta-analyses.

Given the narrative nature of this review, a formal quantitative synthesis was not performed. However, key methodological elements were predefined to enhance transparency and reproducibility, in line with the core principles of the PRISMA recommendations.

## 3. Beneficial Effects of Exercise Training in PAD: Mechanistic Overview

### 3.1. Endothelial Function and Nitric Oxide (NO)

Intermittent ambulatory exercise increases pulsatile and mean shear stress across conduit and resistance vessels, activating endothelial NO synthase (eNOS) and improving vasodilator reserve. This shear stress-dependent mechanism is a central pathway through which exercise exerts favorable effects on endothelial function and vascular adaptation [7]. Exercise also modulates oxidative stress and enhances antioxidant enzyme expression, further preserving NO bioactivity and reducing endothelial dysfunction [8].

Mechanistically, the hemodynamic stimulus of repeated exercise bouts increases endothelial shear stress, upregulates eNOS expression and phosphorylation, and enhances NO release, while exercise-induced upregulation of antioxidant defenses mitigates NO degradation by reactive oxygen species, collectively improving vasodilator capacity [9].

**Figure 2:**
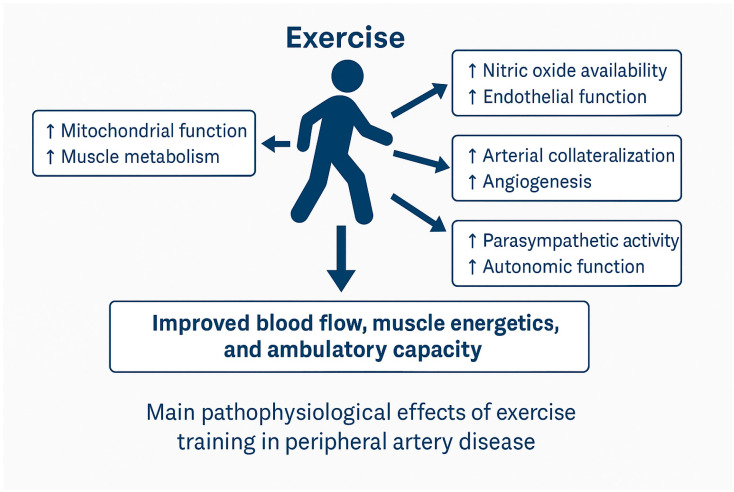
Main pathophysiological effects of exercise training in peripheral artery disease. Repeated ischemia–reperfusion during structured walking and adjunct modalities (e.g., cycling and resistance training) increases endothelial shear stress (↑eNOS/NO; improved brachial FMD), stimulates collateral vessel growth and capillarization (↑VEGF signaling), improves mitochondrial function and oxidative metabolism in ischemic skeletal muscle (↑PGC 1α–driven biogenesis; delayed lactate accumulation), and rebalances autonomic control (↑parasympathetic tone; improved HRV). These adaptations translate into higher pain free and maximum walking distances, better 6 min walk performance, modest ABI gains, and improved quality of life. Importantly, they complement risk factor modification and comprehensive cardiac rehabilitation.

Brachial artery flow-mediated dilatation (FMD) is widely used as a surrogate for endothelial function and is predictive of cardiovascular risk [10], and each 1% decrement in FMD is associated with an approximately 8–13% increase the risk of future cardiovascular events [11]. Meta-analytic evidence in PAD specifically shows that structured exercise improves brachial artery FMD, while broader cardiovascular populations corroborate exercise-induced FMD gains [12,13] (Figure 2).

### 3.2. Angiogenesis and Collateralization

Repeated ischemic stimuli during exercise trigger a cascade of pro-angiogenic mediators, including vascular endothelial growth factor (VEGF), fibroblast growth factor (FGF), and hypoxia-inducible factor 1α (HIF-1α), which promote capillary rarefaction reversal, and stimulate the formation of collateral vessels [14].

Experimental work in skeletal muscle, including single-cell transcriptomic analysis in animal models, indicates that specific subsets of endothelial cells are metabolically primed for angiogenesis. Endothelial cells expressing high levels of transcription factors such as ATF3/4, enriched in oxidative muscle regions, exhibit greater angiogenic potential and proliferative responses to exercise stimuli, possibly through metabolic regulation of amino acid uptake and vascular expansion pathways [15]. While quantifying human collaterals non-invasively is challenging, improved treadmill and 6 min walk performance after SET is consistent with physiological flow redistribution and microvascular remodeling [16] (Figure 2).

### 3.3. Skeletal Muscle Metabolism and Mitochondria

PAD muscle exhibits mitochondrial dysfunction, fiber type shifts, and impaired oxidative phosphorylation. Exercise training (particularly walking to moderate–severe claudication) enhances mitochondrial biogenesis (↑PGC-1α), oxygen extraction, and lactate kinetics—delaying pain onset and increasing endurance. Functional gains (PFWD, MWD, and 6MWD) observed across trials reflect these peripheral adaptations, even when ABI changes are modest [16,17] (Figure 2).

### 3.4. Autonomic Balance and Baroreflex

PAD is often accompanied by sympathetic predominance and blunted heart-rate variability. Exercise training favorably modulates autonomic control and baroreflex function, which correlates with improved ambulatory capacity and may contribute to systemic risk reduction [18] (Figure 2).

### 3.5. Inflammation and Oxidative Stress

Exercise exerts anti-inflammatory and antioxidant effects.

Chronic inflammation is a key driver of atherosclerosis [19]. Pro-inflammatory cytokines such as interleukins (ILs) and tumor necrosis factor (TNF)-α contribute to vascular dysfunction by triggering endothelial activation via NF-κB signaling [20], promoting eNOS uncoupling [21], and increasing the expression of adhesion molecules that facilitate monocyte attachment and differentiation into macrophages [20,22]. These processes further enhance platelet aggregation and coagulation, ultimately accelerating atherosclerotic progression, with C-reactive protein (CRP) implicated in PAD development and severity [23].

In patients with PAD, exercise showed reductions in CRP or IL-6 [24,25].

A meta-analysis in PAD found significant FMD improvement without parallel reductions in CRP/IL-6/TNF-α, suggesting that endothelial benefits can occur independently or precede measurable systemic inflammatory change, possibly reflecting early or localized vascular adaptations [12] (Figure 2).

## 4. Supervised Exercise Therapy (SET): The Gold Standard

### 4.1. Protocol and Implementation

Guidelines advise structured, intermittent walking aiming for moderate–severe claudication (3–4/5 pain scale) before resting, 30–45 (up to 60) minutes per session, at least three days/week, and over 12 weeks or longer. Programs should individualize treadmill grade/speed, monitor hemodynamics and symptoms, and embed education and risk-factor management. The CMS covers up to 36 sessions (with potential extensions), facilitating program expansion, although copays and access remain barriers [2]. Key trials, meta-analyses, and real-world studies on exercise therapy in PAD are reported in Table 1.

### 4.2. Effectiveness

Randomized trials and meta-analyses consistently show SET improves maximal and pain-free walking distances and 6 min walk distance versus usual care. Notably, in the CLEVER study (aortoiliac disease), six months of SET improved peak walking time more than primary stenting; at 18 months, both SET and stenting remained superior to medical therapy alone, with functional outcomes broadly comparable [27]. Systematic umbrellas confirm robust effects on walking and quality of life [17,27,39].

### 4.3. Endovascular Therapy Plus SET vs. SET Alone

The ERASE trial randomized patients with claudication to selective endovascular revascularization plus SET versus SET alone. At 12 months, the combination achieved greater improvements in MWD and PFWD, with favorable cost-effectiveness from societal perspectives—supporting the “exercise-first, revascularize-when-appropriate” paradigm and highlighting synergy when anatomy and symptoms warrant [28,29].

### 4.4. HIIT and Alternative Intensities Within SET

Although high-intensity interval paradigms can improve endothelial function in cardiometabolic populations, PAD trials emphasize ischemia-inducing walking as the stimulus most consistently linked to functional gains. The LITE trial found that home-based low-intensity walking (avoiding symptoms) did not improve 12-month 6MWD compared with high-intensity walking that provoked leg symptoms. Supervised protocols should therefore target symptom-limited intensities with careful monitoring [31].

## 5. Home-Based and Hybrid Exercise Models

### 5.1. Why We Need Them

Despite reimbursement, SET uptake remains low. Early Medicare data (2017–2018) showed only ~1.3% of beneficiaries diagnosed with claudication participated in SET; most did not complete all sessions. Barriers include limited program availability, copayments, travel/time, and low provider referral rates. Surveys in urban PAD cohorts confirm physician non-referral and copays as common obstacles [40,41].

### 5.2. Safety of Home-Based Programs

A systematic review of 27 studies (147,810 patient-hours) reported only four related adverse events (≈1 per 36,953 h), three cardiac, mostly occurring when walking to high pain levels; safety appears greater with pre-exercise cardiac screening. Importantly, structured, monitored home programs differ from unstructured “go home and walk” advice [35].

### 5.3. What Works at Home

McDermott’s GM-CBT trial demonstrated that a structured home-based walking program using group-mediated cognitive behavioral strategies improved 6MWD by ~53 m at six months vs. attention control, with gains in patient-perceived walking ability and physical activity.

Recent large randomized data confirm that structured home-based walking exercise significantly improves 6 min walk distance compared with control, with adherence unaffected by age, sex, or baseline functional status, although certain comorbidities may influence adverse events [34].

Conversely, a trial relying primarily on wearable monitoring plus telephone coaching (HONOR) showed no improvement, underscoring that behavioral structure and periodic in-person support drive efficacy [26,42]. A recent meta-analysis demonstrated that home-based exercise interventions significantly improve both pain-free walking distance and maximal walking distance compared with control groups, with clinically meaningful effects on walking performance [38].

### 5.4. Digital and Hybrid Designs

Contemporary randomized data (e.g., WalkingPad RCT) show that adding app-supported behavior change to prescribed home-based walking can improve PFWD/MWD and quality of life versus prescription alone. Hybrid models that begin with brief supervised phases and transition to monitored home training (e.g., HY-PAD) have shown high adherence and clinically meaningful functional gains, offering scalable pathways where SET capacity or patient logistics are limiting [36].

Extending this behavioral paradigm, the GAMEPAD trial demonstrated that gamification combined with automated coaching significantly increased daily ambulatory activity compared with wearable monitoring alone, demonstrating that behavioral economic strategies can enhance ambulatory activity in PAD [43]. Whether these activity gains translate into durable improvements in functional performance and clinical outcomes warrants further study.

### 5.5. Real-World Outcomes and Program Completion

Within a large health system’s first five years of CMS coverage, just 773/5320 PAD patients were referred and 415 enrolled; only ~50% completed SET, yet completers achieved significant functional and quality-of-life improvements. Such findings reinforce the need for system-level referral prompts, copay mitigation, and integrated vascular/cardiac rehabilitation teams [44].

## 6. Alternative and Adjunct Exercise Modalities

### 6.1. Resistance Training

Lower-limb resistance training increases strength and may improve walking capacity; meta-analyses suggest meaningful effects on strength, with variable transfer to walking compared with treadmill-based SET. When orthopedic limitations or severe pain preclude walking, resistance sessions (2–3×/week, targeting major muscle groups, with 1–3 sets of 8–12 reps at moderate intensity) can be used as a bridge or adjunct [17].

### 6.2. Arm Ergometry (Arm Crank)

Upper-body endurance training can elicit cross-transfer benefits to leg function by improving systemic cardiovascular fitness and autonomic regulation when lower-limb ischemia limits adherence—useful particularly in early phases or for patients awaiting revascularization [45,46].

### 6.3. Cycling

Stationary cycling is often better tolerated than treadmill walking and improves cardiorespiratory fitness, though walking-specific improvements are generally smaller than with SET because the ischemic stimulus to calf musculature is less targeted. Cycling can complement walking in order to accumulate aerobic minutes within symptom tolerances [16].

### 6.4. Hydrotherapy

Small controlled studies report that combining a supervised program with lower-limb whirlpool massage can improve impedance plethysmography indices and 6MWD versus exercise alone—hypothesized via vasodilation and reduced peripheral resistance—though the evidence base remains early and requires replication [47,48].

## 7. Clinical Outcomes

### 7.1. Functional Performance

Across RCTs and meta-analyses, SET produces large improvements in MWD (~120–200 m or more), PFWD, and 6MWD, with clinically meaningful changes that exceed those achieved by usual care and often rival those after revascularization at medium-term follow-up. Patient-reported walking impairment domains (distance, speed, and stairs) also improve substantially [17,27,49,50].

### 7.2. Endothelial Function

Exercise training increases brachial FMD in PAD; mechanistic work suggests benefits are optimized at moderate–vigorous intensities and accrue across diverse cardiometabolic states. Given the prognostic value of FMD, these vascular adaptations may represent an important pathway linking exercise to event risk reduction [12,13,51,52].

### 7.3. Quality of Life (QoL)

Both SET and revascularization improve disease-specific QoL. In CLEVER, certain QoL scales favored stenting early while treadmill performance favored SET, indicating complementary roles. Combination strategies (ER+SET) can provide larger short-term gains where anatomy and symptoms justify intervention [27,28].

### 7.4. ABI and Hemodynamics

ABI may rise modestly in some trials, yet functional improvement frequently exceeds hemodynamic change, reflecting dominant peripheral adaptations. Clinicians should therefore prioritize symptom-targeted training and function-based outcomes rather than ABI alone [16].

### 7.5. Mortality and Hospitalizations

Although PAD exercise trials are typically underpowered for mortality, participation in comprehensive cardiac rehabilitation programs is associated with improved survival and risk-factor control in PAD cohorts. Observational evidence suggests that SET may reduce the subsequent need for endovascular/surgical procedures compared with non-participants [40,53,54].

## 8. Prehabilitation and Post-Revascularization Rehabilitation

Prehabilitation—4–8 weeks of structured training before planned interventions—aims to improve functional reserve and perioperative resilience. Post-revascularization, combining SET with guideline-directed medical therapy improves walking distances more than either strategy alone and may reduce repeat interventions by addressing the systemic and peripheral drivers of exertional limitation [2,28,55,56,57].

## 9. Safety, Risk Stratification, and Contraindications

SET is safe when delivered under direct supervision with appropriate screening and monitoring; adverse events are rare. For home-based programs, systematic review evidence indicates very low event rates, with most complications occurring when patients push to severe pain intensities and in programs lacking cardiac screening—hence the importance of initial evaluation, progressive loading, and clear stop rules. Absolute contraindications mirror those in general cardiac rehabilitation (e.g., unstable angina, decompensated heart failure, and critical limb ischemia with infection) [2,37,58].

## 10. Patient Education and Communication

Engagement in exercise-based rehabilitation for PAD is fundamentally influenced not only by the exercise prescription itself but also by patient education, communication strategies, and behavioral support. Adherence to exercise programs remains a critical determinant of efficacy, yet real-world data indicate suboptimal participation and completion rates, particularly for SET. The emerging literature suggests that addressing educational needs and motivation may improve adherence and functional outcomes.

Understanding patient barriers and motivations is essential: many individuals with PAD face logistical challenges (transportation and time constraints), misconceptions about pain and exercise safety, and lack of confidence in self-managed programs. Structured education interventions that address these factors can empower patients to participate and sustain activity over time.

Randomized trials incorporating behavioral and educational components underscore the value of this approach. For example, group-mediated cognitive behavioral HBET strategies, such as those used in the GOALS trial, significantly improved 6 min walk distance and physical activity levels compared with control conditions, highlighting how education and group support can enhance adherence and outcomes in home-based settings [26]. Similarly, behavior-change interventions delivered by trained therapists (e.g., walking exercise behavior change versus usual care) have been shown to improve 6 min walk distance over short-term follow-up periods, suggesting that motivational communication strategies can facilitate engagement, even in clinical populations with intermittent claudication [33].

A growing body of evidence also supports the integration of technology to augment education and communication. Smartphone-enabled tools and mobile apps that provide motivational messages, progress tracking, and education on goals and symptoms have been proposed as promising adjuncts to conventional programs, with trial protocols such as the WalkingPad study explicitly incorporating educational and motivational intervention components [59]. Although definitive results from these technology-driven strategies are still forthcoming, they exemplify how patient communication can be embedded within exercise regimens to support self-management and adherence.

Observational evidence and scoping reviews further reinforce the importance of communication and educational support in routine care [60]. Barriers to implementation of best-practice exercise protocols often include insufficient patient education about the benefits and safety of exercise, inconsistent reinforcement of goals, and limited provider communication about how to manage symptoms during activity.

Taken together, these data suggest that patient education and structured communication should be considered core components of exercise therapy for PAD. Effective strategies include personalized counseling on symptom management and exercise goals, behavioral support to address motivation and self-efficacy, use of group support or peer engagement, and, where feasible, incorporation of digital tools to reinforce adherence and provide ongoing feedback. Future research should continue to evaluate the optimal content, delivery methods, and timing of educational interventions within PAD rehabilitation to maximize both adherence and clinical outcomes.

## 11. Implementation and Policy

Despite coverage and strong guidelines, SET referral and completion are modest: in early national data, only ~1.3% of eligible Medicare beneficiaries enrolled within 19 months of coverage, copays and travel/time logistics were key barriers, and most referring clinicians had not prescribed SET. Health system registries confirm low referral rates but meaningful gains among completers. Addressing copays, embedding referral prompts in vascular/cardiology clinics and co-locating PAD programs within cardiac rehabilitation are pragmatic steps forward [40,41,44]. The 2024 ESC/ESVS consensus and ESVS guideline for asymptomatic PAD/claudication emphasize supervised or structured community/home-based programs as core therapy, while acknowledging unmet needs and disparities across Europe. This converges with 2024 ACC/AHA guidelines, which elevates structured exercise alongside pharmacotherapy and selective revascularization [16,61].

## 12. Practical Exercise Prescription in PAD (SET or Structured Home Programs)

Assessment and baseline: ABI with waveforms, symptom profile, risk amplifiers (diabetes, CKD, and smoking), fall risk/orthopedic issues; consider baseline 6MWT and/or graded treadmill test to individualize speed/grade and monitor calf muscle StO_2_ if available [2].

Aerobic component (walking centered): 3–5 days/week; warm-up 5–10 min; walk at a speed/grade that brings on moderate–severe claudication within 3–5 min; continue walking until 3–4/5 pain, then rest until symptoms resolve; repeat cycles to total 30–45 min; cool-down 5–10 min. Progress by increasing grade/speed or reducing rest intervals as tolerated; seek ischemic stimulus but avoid maximal, intolerable pain [2].

Adjunct modalities: Cycle ergometry or arm crank on non-walking days to accumulate 150–300 min/week moderate-to-vigorous aerobic activity if feasible; add 2–3 days/week of resistance training targeting lower limbs to augment strength, balance, and daily function [16,17].

Behavioral scaffolding for home or hybrid: Use pedometers/accelerometers, app-supported goal setting, and scheduled coaching; consider a brief supervised phase to establish intensity and technique, then transition to monitored home training with periodic in-person reassessments [35].

Safety tips: Educate on red flags (chest pain, syncope, new rest pain/wounds, and infection); consider pre-exercise cardiac screening in higher-risk patients; titrate intensity to symptom targets; maintain foot care and appropriate footwear; and ensure hydration and medication adherence [37].

## 13. Special Populations

Older adults and frailty: Prioritize safety, balance, and progressive loading; consider seated/recumbent modalities early. Even small functional gains can translate into meaningful independence improvements [16,62].

Diabetes and microvascular disease: Glycemic variability influences walking tolerance; careful glucose monitoring around sessions and foot surveillance are critical. Exercise also improves endothelial function in diabetes, supporting its inclusion as a vascular health intervention [63].

Women and socioeconomically disadvantaged groups: Epidemiology indicates disproportionate impacts and access barriers; flexible scheduling, transportation support, and copay assistance can help close gaps in participation [5,64,65].

## 14. Clinical Pathway for PAD Management in CR

A clinical pathway for PAD management in CR is proposed:

(1) Confirm diagnosis and phenotype (asymptomatic, claudication, and CLTI) with ABI and targeted testing;
(2) Initiate guideline-directed medical therapy (statins, antiplatelets, and smoking cessation; consider rivaroxaban 2.5 mg bid + aspirin in appropriate patients for MACE/MALE risk reduction);
(3) Enroll in SET (or structured hybrid/home if SET access limited), delivering a progressive, symptom-targeted walking program with risk-factor management;
(4) Reassess at 12 weeks: If insufficient improvement and anatomy is amenable, consider selective endovascular/surgical revascularization, with post-procedure rehabilitation to consolidate gains;
(5) Long-term maintenance through community programs, digital tools, and periodic supervised “boosters”.

## 15. Unresolved Questions and Research Priorities

Despite robust evidence supporting structured exercise therapy as first-line treatment for claudication, several critical gaps remain in our understanding of how exercise should be optimized, personalized and integrated into comprehensive PAD care.

Most randomized trials of supervised or home-based exercise in PAD have been powered for functional endpoints such as maximal walking distance or 6 min walk distance, with limited data on major clinical events. Although increased physical activity is associated with lower cardiovascular mortality in PAD cohorts, definitive evidence that structured exercise independently reduces MACE or MALE is lacking [2]. Prospective, event-driven studies are needed to determine whether exercise confers incremental prognostic benefit beyond guideline-directed medical therapy and whether functional improvements translate into reduced hospitalizations, revascularization procedures, amputation, or mortality, particularly in high-risk subgroups (e.g., people with diabetes or polyvascular disease) where mechanistic benefits have been inferred but not directly linked to clinical endpoints.

Moreover, although walking to moderate–severe claudication pain has traditionally been considered the most effective stimulus for improving walking performance in PAD, emerging evidence suggests that the optimal training intensity may vary substantially across individuals and according to the outcomes prioritized. The LITE (Low-Intensity Exercise Intervention in PAD) trial suggests a potential paradox in community-based exercise therapy for PAD, as patients who walk through pain often achieve greater functional improvements but limited gains in quality of life, whereas those who avoid pain may report better quality of life despite smaller functional improvements [31]. Also, a network meta-analysis showed variable effects of exercise intensity (high-pain vs. low-pain protocols) on functional outcomes [66].

These findings suggest that the efficacy of exercise therapy in PAD cannot be defined solely by objective functional metrics, but should also incorporate patient-reported outcomes, including perceived walking ability, symptom burden, and quality of life. In clinical practice, exercise prescriptions should therefore be considered dynamic and individualized, with training intensity titrated according to symptom tolerance, functional response, and patient priorities.

Systematic integration of patient-reported outcomes alongside objective measures may facilitate a more patient-centered evaluation of treatment effectiveness and guide the ongoing adjustment of exercise prescriptions.

Emerging pharmacological strategies, including newer antithrombotic agents, have been highlighted as central to the management of PAD due to the high thrombotic and limb event risk observed in this population, underscoring the importance of tailored antithrombotic approaches in daily practice [67]. PCSK9 inhibitors, SGLT2 inhibitors, and GLP-1 receptor agonists possess established cardiovascular and metabolic benefits, and narrative reviews in PAD suggest potential effects on limb outcomes and systemic risk, though evidence remains controversial and limited.

PCSK9 not only regulates LDL-C metabolism but also participates in vascular pathological processes and correlates with PAD severity, suggesting potential roles in disease modification beyond lipid lowering [68]. Meta-analyses in PAD indicate that PCSK9 inhibitors significantly reduce LDL-C, major adverse cardiovascular events, major amputations, and myocardial infarction in high-risk PAD populations, although their effects on vascular hemodynamic parameters (e.g., ABI and FMD) are less clear [69]. Furthermore, pharmacotherapies such as SGLT2-i and GLP-1RA exhibit effects on inflammatory pathways and plaque biology, indicating a mechanistic rationale for benefits beyond glycemic control, but more clinical data are needed to determine their interaction with structured exercise interventions in PAD [70].

Furthermore, emerging digital technologies offer opportunities to enhance personalization and monitoring of exercise programs. Artificial intelligence (AI) can support adaptive exercise prescriptions and risk stratification based on real-time patient data, while 3D printing, although primarily procedural in vascular surgery, exemplifies the broader trend toward precision medicine that could eventually inform patient-specific rehabilitation strategies [71,72].

Future studies should aim to define individualized intensity thresholds, integrate real-time symptom monitoring and patient-reported outcomes, and determine whether adaptive exercise strategies can improve both adherence and long-term clinical outcomes in PAD rehabilitation.

## 16. Conclusions

Exercise training is the therapeutic linchpin for claudication: it addresses the peripheral problem (ischemic muscle energetics), vascular dysfunction (endothelial/angiogenic), and systemic risk (autonomic balance and fitness), delivering durable improvements in walking ability and quality of life. SET remains the gold standard where available; structured, monitored, home or hybrid programs are safe and effective solutions to reach more patients. Embedding exercise within comprehensive cardiac rehabilitation, reducing copays, and expanding referral pathways are essential to translate the science into routine care for the millions living with PAD.

## Figures and Tables

**Table 1:** Key trials, meta-analyses, and real-world studies on exercise therapy in PAD.

Study (Year)	Population/Setting	Arms/Intervention	Primary Outcome	Key Results	Follow-Up	Notes
GOALS (2013) [26]	PAD (IC + non-IC)	HBET (group-mediated cognitive behavioral) vs. Control	6MWD; treadmill walking; WIQ	HBET improved 6MWD by +53.5 m vs. control; improved treadmill time & WIQ	6 mos.	Classic RCT for HBET programs
CLEVER (2013) [27]	Aortoiliac PAD with IC	OMC vs. Stent vs. SET	Peak walking time (graded treadmill); QoL	SET > Stent at 6 mos. for treadmill performance; both SET and Stent > OMC at 18 mos.; QoL favored Stent early	6 and 18 mos.	Functional vs. QoL trade-offs; importance of maintenance
ERASE (2015; 2021) [28,29]	IC (aortoiliac/femoropopliteal)	SET vs. Endovascular + SET	MWD	+282 m with combination vs. SET alone; cost-effective from societal perspective	12 mos.	Supports synergy when anatomy warrants intervention
Lane et al. (2017) [30]	IC (meta-analysis of 32 RCTs)	SET vs. OMC	PFWD; MWD; ABI; QoL; mortality/amputation	SET improved PFWD and MWD (MD ≈ 82–120 m). No effect on ABI, mortality, or amputation. Some QoL domains improved	2 wks. to 24 mos.	moderate heterogeneity; high-quality evidence for walking distance improvements;
LITE (2021; 2025) [31,32]	PAD with diverse symptoms	High-intensity (symptom-eliciting) HBET vs. Low-intensity (pain-free) HBET vs. Control	6MWD; PROMs	High-intensity +34–45 m at 12 mos.; low-intensity no benefit vs. control; PROMs improved mainly with high-intensity	12 mos.	Coaching weekly; accelerometer-monitored
MOSAIC (2022) [33]	PAD with IC	HBET vs. Control	6MWD	HBET improved 6MWD by +16.7 m vs. control at 3 mos.	3 mos.	Multicenter RCT. Walking exercise with motivational behavior change delivered by physical therapists improved walking distance; further durability research needed
Thangada et al. (2025) [34]	PAD RCTs (n ≈ 719)	HBET vs. SET treadmill vs. controls	6MWD and treadmill outcomes	HBET > SET for 6MWD (+≈24 m); SET > HBET for treadmill distance	6 mos.	Provides head-to-head evidence; supports HBET first-line
Silva et al. (2023) [35]	IC; single-center	HBET + behavior change ± smartphone app	PFWD; MWD; 6MWD; QoL	Both arms improved at 3 mos.; MWD advantage with app at 6 mos. in sensitivity analyses	3–6 mos.	Digital augmentation potentially helpful
HY-PAD feasibility (2025) [36]	PAD; pre–post	4 wks. SET then 8 wks. HBET with calls	6MWD; WIQ	+56 m 6MWD; high adherence; few adverse events	12 wks.	Feasibility; needs controlled trials
Waddell et al. (2022) [37]	IC; 27 studies; 147, 810 patient-hours	HBET (varied)	Complication rate	≈1 related event/36,953 patient-hours; most without cardiac screening	Varied	Supports safe HBEP with prudent screening
Xu et al. (2025) [38]	IC; 8 RCTs	HBET vs. control	PFWD/MWD	HBET improved PFWD and MWD (SMD ~0.47–0.67)	6–52 wks.	Intensity/adherence likely moderators. High heterogeneity
Lee et al. (2024) [12]	1301 PAD participants (meta-analysis of 18 studies; some with CAD comorbidities)	Structured aerobic training (SET/HBET)	FMD; inflammatory biomarkers	Increased brachial FMD following training; No significant change in inflammatory biomarkers	Varied (mean 17 wks.; range 6–40 wks.)	Mechanistic support for vascular benefit

Abbreviations: 6MWD—6 min walk distance; FMD—flow-mediated dilation; HBET—home-based exercise therapy; IC—intermittent claudication; MWD—maximal walking distance; OMC—optimal medical care; PAD—peripheral artery disease; PFWD—pain-free walking distance; PROMs—patient-reported outcome measures; QoL—quality of life; SET—supervised exercise therapy; WIQ—Walking Impairment Questionnaire.

## Data Availability

No new data were created or analyzed in this study.
